# Constructing a Nanopipette-Based DNA Electromechanical Device

**DOI:** 10.1021/acs.nanolett.5c05156

**Published:** 2025-12-15

**Authors:** Cengiz J. Khan, Oliver J. Irving, Rand A. Al-Waqfi, Giorgio Ferrari, Tim Albrecht

**Affiliations:** ‡ School of Chemistry, Edgbaston Campus, 1724 University of Birmingham, Birmingham B15 2TT, United Kingdom; § Department of Physics, 274268 Politecnico di Milano, Piaza L. da Vinci 32, Milano 20133, Italy

**Keywords:** nanopipettes, DNA nanotechnology, nanoparticles, trapping, nanoelectromechanical

## Abstract

Solid-state nanopore and nanopipette sensors are powerful devices for the detection, quantification, and structural analysis of biopolymers such as DNA and proteins, especially in carrier-enhanced resistive-pulse sensing. However, hundreds of different molecules typically need to be sampled from solution and analyzed to obtain statistically robust information. This limits the applicability of such sensors and complicates associated workflows. Here, we present a new strategy to trap DNA structures in the sensing region of a nanopipette through end functionalization and nanoparticle capping. We develop a robust set of descriptors to characterize the insertion and presence of nanoparticle–DNA constructs in the nanopipette tip and show that they remain mobile and responsive to external electric fields over extended periods of time. This is for repeated readout of the same DNA structure and could enable new applications for such sensors, for example, in flow and in confined environments.

## Graphical abstract

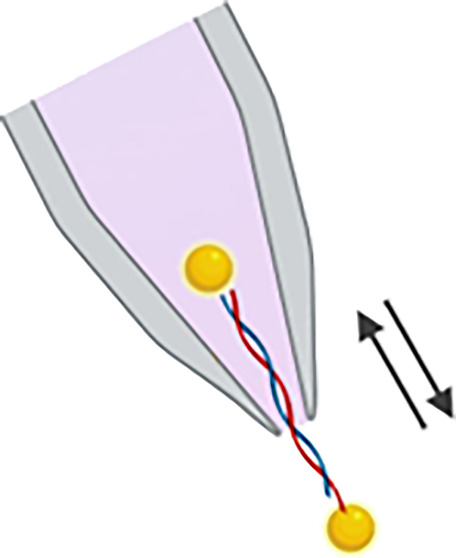

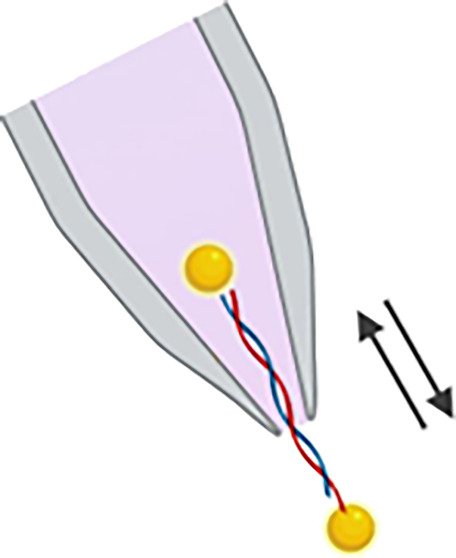

Nanopore sensors are powerful tools for the study of small-molecule transport through protein nanochannels and the detection of nanoparticles, DNA, and RNA as well as proteins. They have transformed DNA and RNA sequencing, with interesting prospects toward protein sequencing as well,^1–5^ and are also compatible with electric, fluorescence, Raman, electrochemical, and quantum-tunnelling-based detection.^6–10^ To this end, electric readout is of particular interest for point-of-care applications, as the small footprint, compatibility with microfluidic sample processing and device miniaturization are advantageous features in this regard.^11–13^ The operating principle of a “resistive-pulse” sensor is relatively simple in that the passage (“translocation”) of suitable analytes such as individual DNA, proteins or particles through the nanopore can result in characteristic events in the current–time signal. Detailed analysis of such events, for example, their frequency, duration, magnitude and substructure, can provide a wealth of information about the analyte in question.^14–17^ This information “richness” opens up interesting new avenues for bioanalytical applications. For example, in carrier-enhanced nanopore sensing, long, kilobase pair (kbp) DNA is functionalized with specific capture probes, such as antibodies, aptamers or oligonucleotides, in well-defined locations.^18–20^ Upon incubation with analytes of interest, subsequent translocation of the carrier DNA can reveal the binding state of each capture probe, thereby confirm the presence of an analyte and provide an estimate of their concentrations.^19^ Furthermore, the ability of nanopore sensors to resolve structural features on the carrier DNA less than 100 nm apart allows for multiplexed detection on a single carrier,^19,21^ which may be further enhanced by mixing different, distinguishable carriers.^21^ Finally, it has been noted that carrier-enhanced sensing may facilitate the detection in more complex mixtures.^22,23^

Typically, several hundred translocation events need to be recorded to build up a sufficiently robust statistical basis.^14–16,24^ These are based on different molecules or particles, unless they are recaptured by fast bias reversal.^16,25,26^

Ultimately, this raises the question whether a functional DNA carrier could be permanently trapped in the sensing region. In this way, sample incubation would only involve target binding to the trapped carrier, and rather than capturing freely dissolved carriers for translocation, the carrier would already be present in the sensing region of the device. Hence, repeated readout of bioanalytical information, for example, by incorporating antibody or aptamer-based capture probes for biomolecular targets along the DNA, could be achieved by applying oscillatory electric fields. Such an approach would be applicable to different classes of nanopore sensors, including chip-based and nanopipettes, and significantly simplify aspects of the sensing workflow. It could also facilitate measurements in flow and, particularly for nanopipettes, in confined spaces, such as individual cells.^7,27,28^

Here, we demonstrate the step-by-step fabrication of such a nanoelectromechanical device (NEMD), by combining DNA engineering, nanoparticle chemistry and electrophoretically driven assembly in a nanopipette, Figure 1. We characterize the assembly systematically, with a combination of biochemical, structural, optical, and electrical methodologies, and demonstrate, based on representative example data sets, the stable formation of as well as the capability for bidirectional transport of the trapped DNA structure.

**Figure 1:**
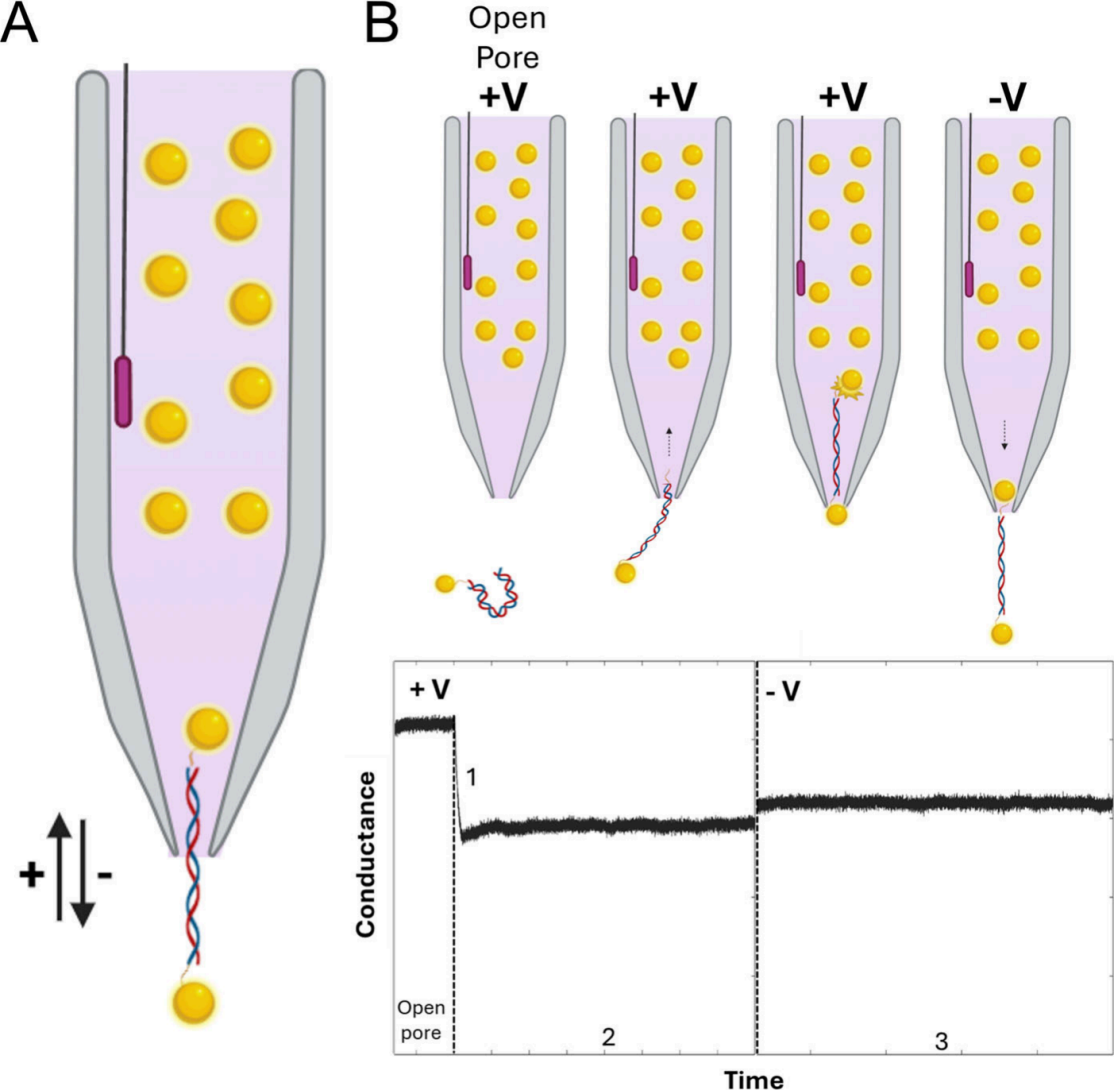
(A) Illustration of the NP/DNA/NP construct trapped in a nanopipette. The application of a bias voltage can drive the structure in or out of the pipet, approximately over the length of the DNA. The NPs are larger than the inner pore diameter of the nanopipette, preventing the escape of the trapped structure. (B) Step-by-step assembly (top) and pore conductance as a function of time (bottom). Initially, the conductance is at the open-pore value. The DNA is modified with an azide group at one end and biotinyl at the other, allowing for orthogonal binding of monofunctionalized DBCO- and streptavidin-modified NPs, respectively. A NP/DNA construct is electrophoretically driven into the pipet (1) and arrested as the NP is unable to translocate. Binding of the counterparticle on the inside of nanopipette completes the NP/DNA/NP construct (2). The open-pore conductance is not recovered upon bias reversal (3), suggesting that the structure can no longer be ejected.

This strategy is based on the idea that the binding of metallic nanoparticles to end-functionalized DNA can provide a “stopper”, preventing the escape of the DNA construct from the sensing region of the nanopipette. In our experiments, we used gold nanoparticles (AuNPs) that had a diameter substantially larger than the inner diameter of the nanopipette tip (∼2:1). Preformed NP/DNA constructs were guided into the nanopipette using externally applied electric fields, an approach inspired by previous work on DNA Origami nanopores.^29^ The second “counter”particle was made available on the inside of the nanopipette, such that the formation of the complete NP/DNA/NP construct could only occur with the inserted NP/DNA complex, Figure 1. The different steps in this process were monitored in real-time using electrical recordings.

We start with the preparation and characterization of the individual device components; cf. Figure 2 and section S1. 5 kbp DNA was prepared using PCR amplification with primers carrying azide- and biotinylated groups at the respective 5′ ends. The final DNA product thus featured two orthogonal binding groups for dibenzocyclooctyne (DBCO)- and streptavidin-modified AuNPs (core diameter = 40 ± 2 nm, Nanopartz, Loveland, CO). Importantly, both types of particles predominantly feature a single binding site, according to the manufacturer’s specifications, thereby facilitating the specific formation of DNA/particle constructs and reducing the probability of oligomerization. Subsequently, different building blocks of the final NP/DNA/NP design were initially prepared in free solution and then characterized by gel electrophoresis (1% agarose, 80 V, 45 min), panel A. Lanes furthest to the left and right contain DNA ladder (GeneRuler DNA Ladder Mix, Thermo Scientific, Waltham, MA).

**Figure 2:**
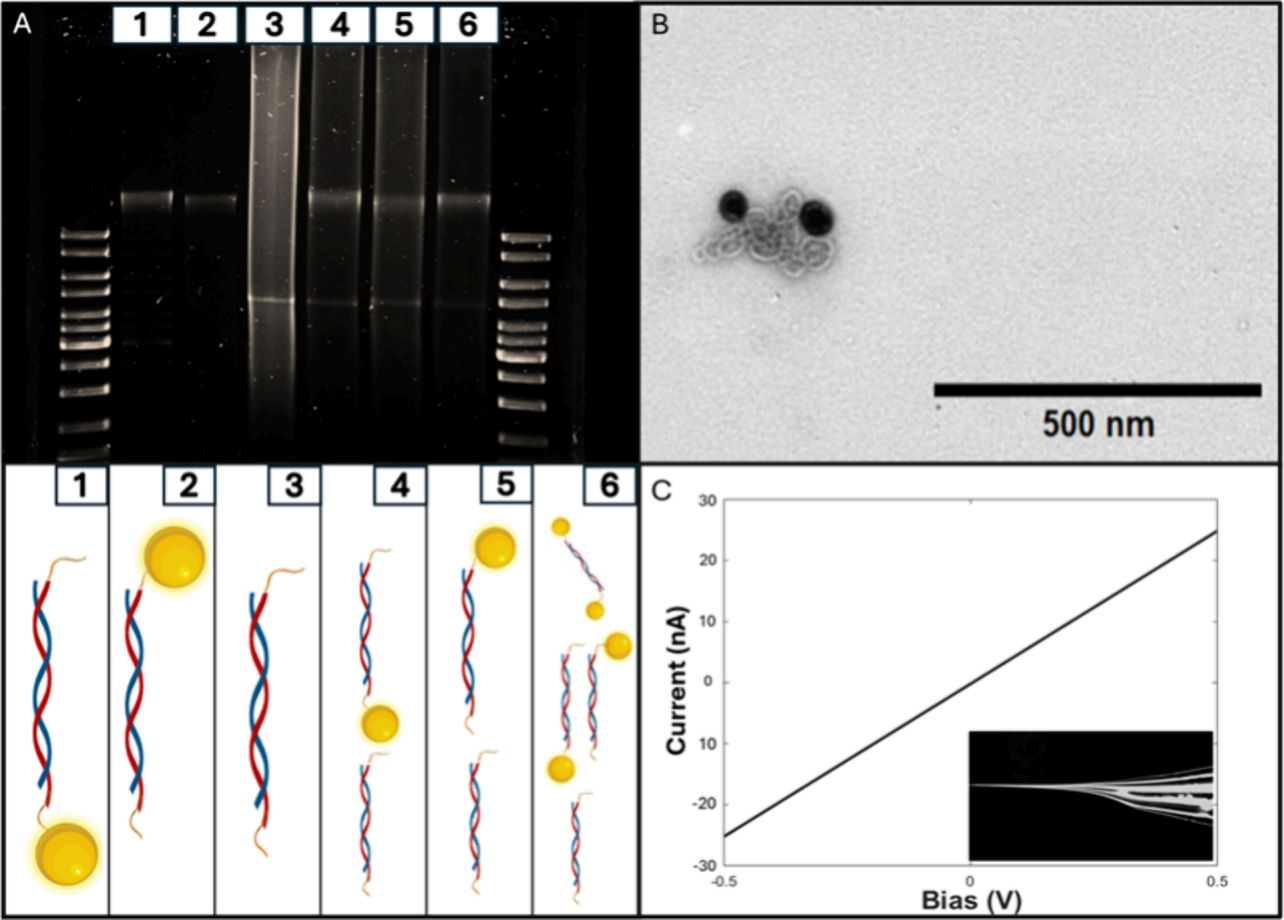
Characterization of the components used to build the dumbbell device: (A) Agarose gel electrophoresis (1% agarose, 80 V, 45 min). Sample 1: DBCO–AuNP bound to 5 kbp DNA. Sample 2: streptavidin-AuNP bound to 5 kbp. Sample 3: unmodified 5 kbp DNA. Sample 4: unpurified reaction mixture of DBCO–AuNP and 5 kbp DNA. Sample 5: unpurified reaction mixture streptavidin–AuNP. Sample 6: unpurified reaction mixture of all components. A schematic of agarose gel well contents can be seen below panel A. (B) TEM image of coiled 5 kbp DNA bound to two AuNPs. (C) Current–voltage trace for a typical nanopipette, 4 M LiCl. Inset: Optical image of the taper.

Lanes 1 and 2 show the DNA conjugated with streptavidin- and azide-modified nanoparticles, respectively, after purification by centrifugation (5000 rpm, 10 min). A single, well-defined band is visible in each case, nominally at DNA lengths >10 kbp. Lane 3 contains the raw PCR product, with the 5 kbp band clearly visible, and lanes 4 and 5 the same samples as in lanes 1 and 2, but before the removal of unbound DNA. Note that the bands for both 5 kbp DNA and conjugate are visible in those lanes. The significant reduction in mobility upon binding of the first particle, regardless of which one, was surprising given the small effect of the particle on the mass and overall charge of the construct. This suggests a more complex interaction between the NP/DNA construct and the gel matrix, as has indeed been observed previously.^30,31^ The sample produced after reacting the DNA with both particles is shown in lane 6. While a faint band corresponding to unreacted 5 kbp DNA still appears, the dominant band is at a similar position as the NP/DNA constructs (lanes 1 + 2). This could either mean that the second particle was bound in insufficient quantities, or that the mobility of the full NP/DNA/NP construct is indeed not significantly different from the NP/DNA complex. We regard the former as unlikely, given the well-known efficiency of avidin/streptavidin and azide/DBCO binding, and note that similar observations regarding the mobility of AuNP–DNA conjugates of varying DNA lengths have been made previously.^31^ Our gel electrophoresis results furthermore confirm that the NPs themselves possess a (small) positive charge (not shown), in accordance with the manufacturer’s specification, suggesting that the mobility of the NP/DNA­(/NP) constructs is dominated by the DNA.

Further support for the successful formation of the NP/DNA/NP construct is provided by TEM imaging, panel B. In this example, both the DNA and the NPs are well-resolved, and suggesting successful binding of both NPs to the DNA; cf. Figure S1.

Nanopipettes were prepared as reported previously, while the pulling parameters were optimized such that the pore diameter was approximately 20 nm (section S4).^14,15^ Accordingly, panel C shows the current–voltage (*IV*) trace of a representative nanopipette chosen from a batch of 10 prepared under the same conditions. For each pipet, the pore conductance, *G*_pore_, was determined from the slope of the *IV* trace between −0.5 and +0.5 V, with an average of 43.5 nS and a standard deviation of 14.0 nS. *G*_pore_ was then used to estimate the (inner) pore diameter, *d*_i_, the nanopipette tip, based on eq 1 in the Supporting Information. Hence, for the pipet shown in panel D, we obtained *G*_pore_ = 47 nS and *d*_i_ = 24 nm.

For the assembly of the NEMD, the preprepared DBCO-NP/DNA complex was provided on the outside of the nanopipette, while the streptavidin-modified NP (strep-NP) was simultaneously present on the inside (electrolyte on both sides, 4 M LiCl; concentration of NP/DNA conjugate, ∼28 pM). A bias of −0.8 V was then applied to drive the NP/DNA complex into the nanopipette, in line with gel mobility results noted above.

Our expectation was that once the DNA part has entered the pore, the structure would be arrested when the NP reached the pore entrance, while the applied (negative) *V*_bias_ reduced the probability of escape in the opposite direction (noting that it is nevertheless subject to Brownian motion). This configuration would allow sufficient time for the strep-NP to bind, thus completing the formation of the NP/DNA/NP construct and trapping it in the nanopipette tip. Importantly, this should lead to sustained reduction in the pore conductance, regardless of bias polarity, since the intact structure can no longer be ejected.

To monitor the above processes in real time, we take advantage of the fact that our setup features two output channels,^14–16^ broadly speaking one containing slow current modulations, including the steady-state pore current (frequency components below *f*_DC_ ∼ 7 Hz, “DC channel”), and the other one fast events, for example, from conventional DNA translocation (frequency components higher than *f*_DC_ up to about 2 MHz, “AC channel”). However, a detailed analysis reveals a more complex relationship between input current modulation and the responses of the individual output channels (section S9). Circuit simulation result show that approximately rectangular pulses in the input current, e.g. originating from short-lived, transient DNA translocation events with a characteristic time τ ≪ 1/2πf_DC_, indeed produce a similar response in the AC channel, while the DC channel remains unchanged. On the other hand, a step-like change in the input current, e.g., from an insertion event of the kind discussed above, produces a more complex response. Initially, it leads to a current modulation in the AC channel, which subsequently returns to its zero-mean value on a time scale of 1/2π*f*_DC_. In parallel, the DC channel evolves from the steady-state current value preinsertion to the new one post insertion, i.e., representative of the nanopore with the trapped structure in place. In other words, insertion events become apparent from transient events in the AC channel and a concurrent, step-like change in the DC channel.

We have therefore expanded the analysis pipeline for the translocation data to include the change in average *I*_DC_, Δ*I*_DC_, and in the AC channel noise, Δσ_AC_, before and after an event (standard deviation, based on 100 ms time); cf. section S5 for more details.

Choosing the Δσ_AC_ in addition to Δ*I*_DC_ was motivated by previous observations that dynamic processes and charge redistribution in the sensing region of the nanopore can lead to an increase in the current noise.^32,33^ Our hypothesis was therefore that trapping of the NP/DNA constructs could result in Δσ_AC_ > 0, while transient occupation of the sensing region (such as for conventional translocation events) should leave the noise level largely unchanged (Δσ_AC_ ≈ 0). This expectation was indeed borne out, as we show below. The noise characteristics of the DC channel were less relevant in this context, as it only captures slow modulations in the input current, as noted above.

We show the results of such an analysis as scatter plots of Δ*I*_e_ vs τ in Figure 3 (*V*_bias_ = −0.8 V; 4 M LiCl, *d*_i_ = 19 nm; cf. Table S2b). The scatter plot in panel A was obtained from a translocation experiment with the PCR product solution, as a control. We have included additional data recorded at different *V*_bias_ in section S5. A distinct cluster of translocation events from the main 5 kbp PCR product is apparent at approximately τ ≈ 0.5 ms and Δ*I*_e_ ≈ 100–200 pA (dashed ellipse). Further analysis of the events in this cluster reveals that those due to linear translocation of DNA are primarily found toward the bottom right of this cluster (the longer τ, the lower Δ*I*_e_), while folded events dominate toward the top left (the shorter τ, the larger Δ*I*_e_), as is well-known from previous work.^14–16^ A more diffuse cluster of events is found between approximately 0.01 < τ < 0.5 ms and 50 pA < Δ*I*_e_ < 200 pA, which is absent in purified DNA samples (not shown).^14–16,34^ We therefore conclude that these events arise from the translocation of DNA byproducts from the PCR reaction. This is in accordance with our gel electrophoresis data, cf. Figure 2A, where smearing due to small nonspecific DNA fragments is apparent. Finally, at τ < 0.01 ms, a distinct cluster due to electric noise emerges. We note that there are essentially no events with τ > 1 ms in this data set. Neither of these event classes feature systematic changes in Δ*I*_DC_ or Δσ_AC_; cf. section S5.

**Figure 3:**
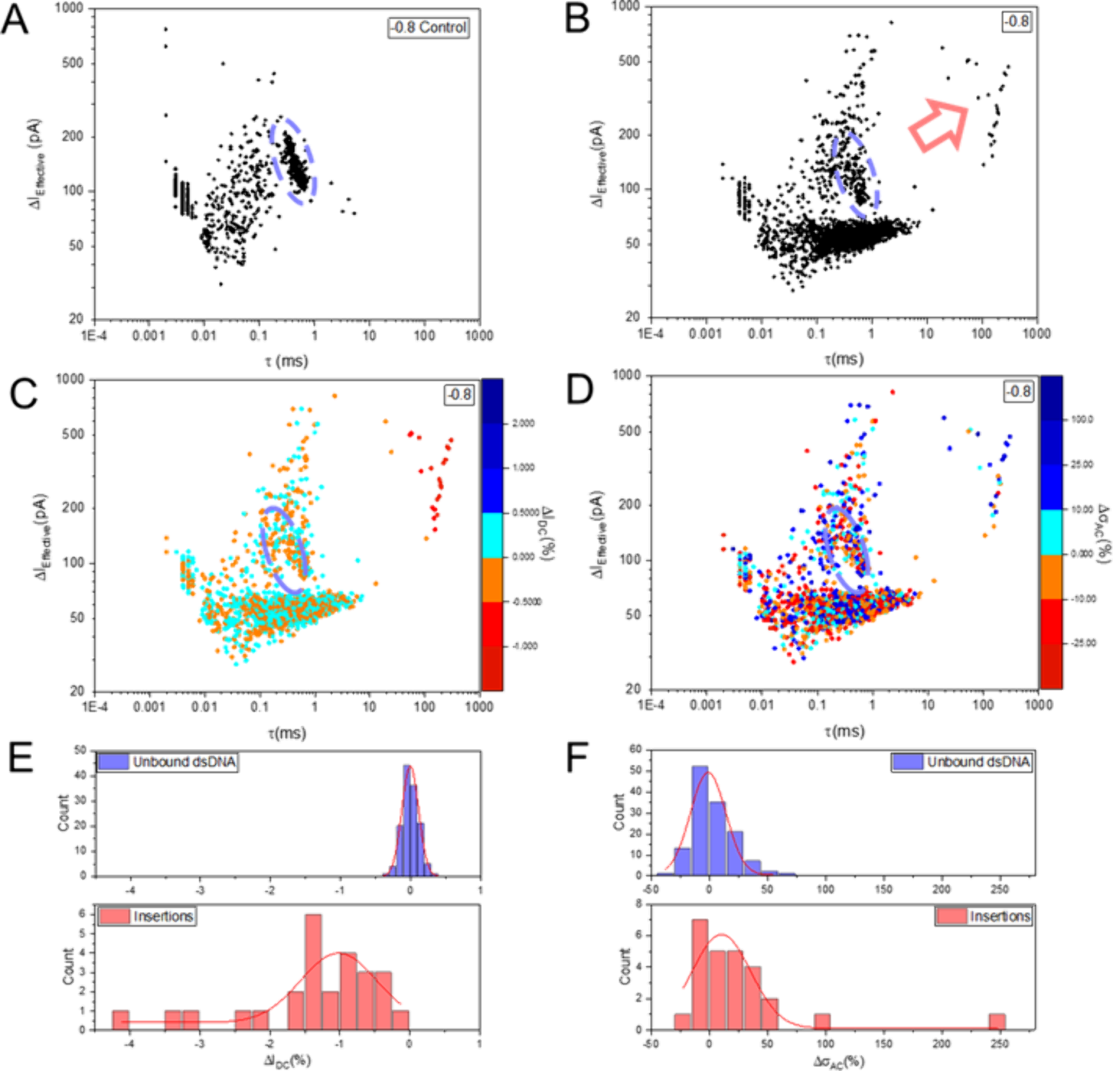
(A) Τranslocation of the PCR solution with (end-functionalized) 5 kbp DNA as the main product as well as shorter byproducts (*V*_bias_ = −0.8 V; 4 M LiCl, *d*_i_ = 19 nm). Very short and low-amplitude events are most likely due to electric noise, while translocation of PCR fragments results in a diffuse cluster with τ below approximately 0.1 ms. Translocation of the 5 kbp DNA results in a well-defined cluster (dashed ellipse). (B) Similar experiment but with NP/DNA complex on the outside and strep-NP on the inside of the nanopipette (*d*_i_ = 24 nm). Results are broadly comparable with those in panel A, but with important differences; see the main text for further discussion. The broad cluster <70 pA is the result of baseline fluctuations. A new cluster emerges at τ ≈ 100 ms and Δ*I*_e_ > 100 pA (red arrow), which we show corresponds to the insertion of NP/DNA complexes. (C) Same data set as in panel B but with Δ*I*_DC_ color-coded (%). Conventional translocation of dsDNA results in an average Δ*I*_DC_ ≈ 0 (see histograms in panel E, top), while insertion events lead to a systematic decrease in *I*_DC_ of about 1% on average (bottom). (D) Same data set as in panel B but with Δσ_AC_ color-coded (in %). Conventional translocation of DNA results in Δσ_AC_ ≈ 0 on average, while insertion events lead to Δσ_AC_ ≈ 20% (panel F).

The same experiment with a similar pipet (*d*_i_ = 24 nm), but now with the products from the NP/DNA assembly reaction on the outside and the respective counter (strep-modified) NP on the inside of the nanopipette. The scatter plot from the analysis of the translocation data, panel B, is in some respects similar to panel A, with a distinct cluster from DNA translocation (dashed ellipse), shorter events from PCR side products as well as electric noise at τ < 0.01 ms. However, there are also important differences. First, we note the dense cluster occurring at low magnitude (<70 pA) over a wide range of τ values 0.01 ms < τ < 10 ms), which we attribute to baseline fluctuations in the presence of the nanoparticles in solution; see also Figure S3d. Second, a small number of events occurs at 0.1 ms < τ < 1 ms with Δ*I*_e_ > 200 pA, which is similar in duration to the 5 kbp DNA PCR product but significantly larger in magnitude; see Figure S5c for representative event shapes. Their exact origin is currently unknown, but it is possible that they originate either from the translocation of NP/DNA complexes (where the NP was too small for trapping) or from insertion and subsequent dissociation of the NP/DNA complex. Third, and more importantly, a small but distinct group of events not present in the control became apparent at τ > 100 ms and 150 pA < Δ*I*_e_ < 500 pA (*N* ≈ 26, red arrow). Their characteristic times were approximately 2 orders of magnitude larger than that for the 5 kbp DNA control, and Δ*I*_e_ somewhat increased.

To better understand their origin, we augment the data shown in panel B with event-wise color-coding of Δ*I*_DC_ and Δσ_AC_ in panels C and D, respectively. It becomes clear that the two event classes—conventional DNA translocation and the long-lived ones at τ > 100 ms—indeed show very different behavior. While the former do not feature systematic changes in Δ*I*_DC_ or Δσ_AC_, see histograms in panels E and F and section S5 for further controls, said long-lived events are characterized by an average Δ*I*_DC_ ≈ −1% (decrease in *I*_DC_ post event) and Δσ_AC_ ≈ +20% (increase in σ_AC_ post event). Both were found to be statistically significantly different at a significance level α = 0.05 (Mann–Whitney, *p* = 4 × 10^–27^ and 0.002, respectively). This is consistent with our expectations for NP/DNA insertion, thereby supporting our interpretation of the physical origin of these events. Those findings are also reminiscent of our recent work, where we found that (reversible) clogging of the nanochannel with 48.5 kbp DNA can lead to a significant decrease in *I*_DC_ and an increase σ_AC_, suggesting that the presence of DNA in the sensing region could be responsible for both.^16^ This would suggest that, in the present case, σ_AC_ may be used as a proxy for the presence of the NP/DNA­(/NP) construct in the pipet tip.

To conclude with some final observations, we have also found that in the presence of the trapped NP/DNA­(NP) structure, conventional translocation of free DNA still continues, albeit at a significantly reduced frequency; cf. Figure S10. This is consistent with the notion that while trapping does not completely block the pore entrance, it decreases its effective area and thus reduces the translocation frequency (of DNA). In some instances, insertion events featured multiple characteristic and transient “dips” in the AC channel current, possibly indicating consecutive interactions between the particle and the nanopipette opening. We show several examples in Figure S5d but abstain from further, more detailed analysis, due to the limited size of the data set.

More importantly, however, to demonstrate successful binding of the NP/DNA complex to the strep-NP counterparticle on the inside of the nanopipette, we investigated the electric characteristics of the sensor post insertion at different bias polarities, as illustrated in Figure 4 A. Our hypothesis here is that the successfully formed NP/DNA/NP construct would no longer be ejected from the nanopipette under reverse bias (*V*_bias_ > 0) and that *G*_pore_ would remain at a value corresponding to the occupied state of the pipet.

**Figure 4:**
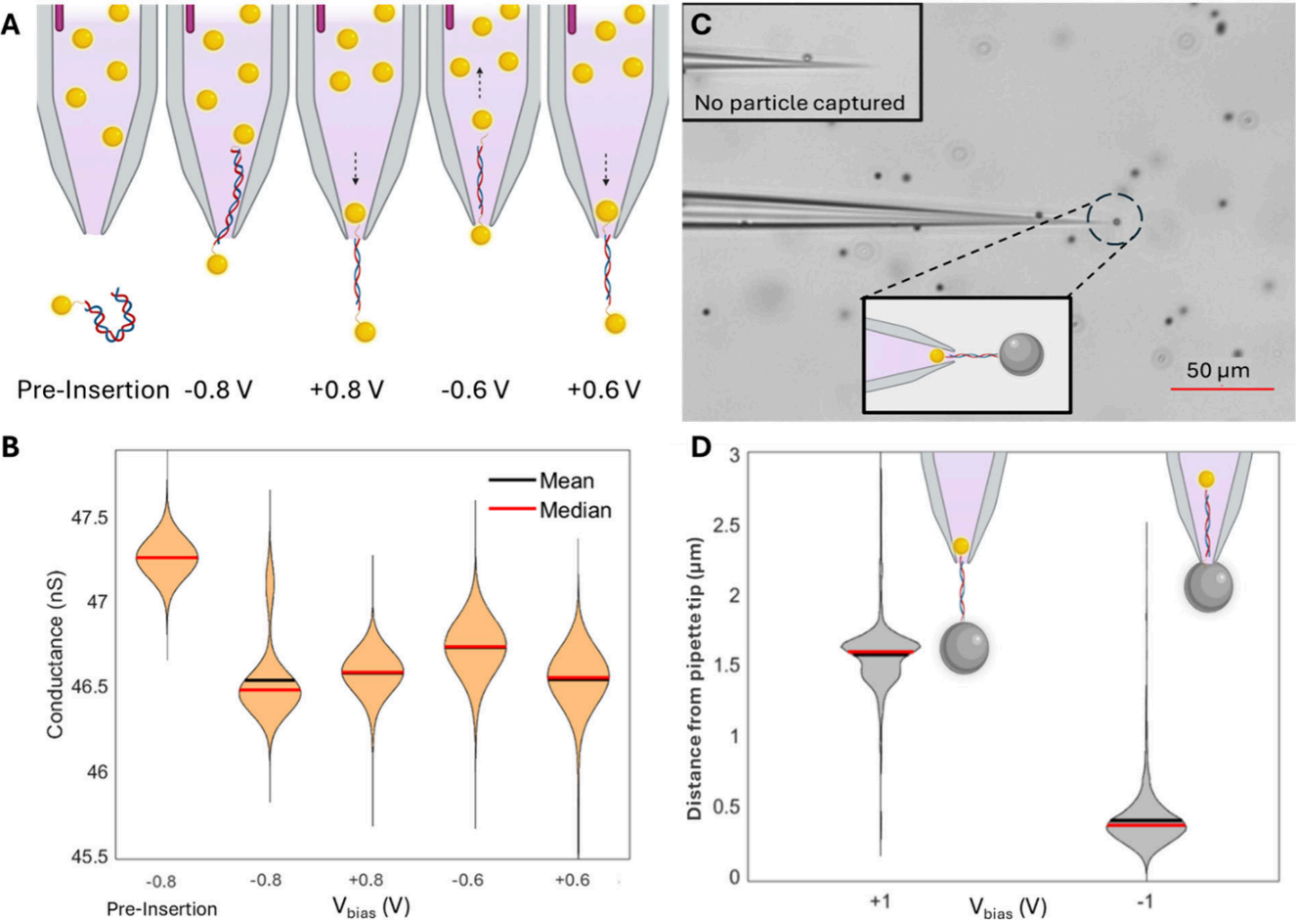
(A) Schematic illustrating the stepwise assembly of the NP/DNA/NP structures and their behavior under bias reversal. All experiments in 4 M LiCl. (B) Violin plots of the conductance for the different stages, from the open-pore (∼47.2 nS, “pre-insertion”) to full assembly under bias reversal. NP-DNA insertion causes a conductance drop (to 46.5 nS), which remains stable under successive bias reversals (+0.8, −0.6, and +0.6 V) over approximately 3000 s. (C) Optical image of a nanopipette with a trapped NP/DNA/MP construct (inset: pipet preinsertion). The MP position is tracked during the different bias conditions (+1 and −1 V) to determine the tether length and stability of the construct. (D) Violin plots showing bead displacement measured from the pipet tip at +1 V (pushed outward) and −1 V (pushed inward). The distributions indicate a tether length of 1.2 ± 0.25 μm. Insets: Schematic depicting bead positions under bias reversal.

This is indeed borne out by the results summarized in Figure 4 B, which shows the probability distributions of *I*_DC_ based on the first 10 s recording time for each *V*_bias_ step (lasting 1000 s each), from “pre-insertion” to *V*_bias_ = −0.8, +0.8, −0.6, and finally +0.6 V.

Note that the drop from the “pre-insertion” value (∼47.2 nS) to the blocked state (∼46.5 nS) occurs during the early stages of step 2 (−0.8 V), leading to a bimodal probability distribution. *V*_bias_ reversal to +0.8 V (step 3), however, does not lead to a recovery of the open pore value and *G*_pore_ remains low at ∼46.5 nS, indicating that the DNA has not been ejected. Correspondingly, *G*_pore_ remains virtually unchanged in steps 3 (*V*_bias_ = −0.6 V) and 4 (*V*_bias_ = +0.6 V), suggesting that the NP/DNA/NP construct has indeed formed successfully and remains in the sensing region of the nanopipette under applied bias conditions for at least ∼3000 s in total.

To further substantiate these findings, we replaced the outside NP by streptavidin-modified polystyrene microparticles (MPs, diameter = 1 μm; Dynabeads, Invitrogen) that can be tracked using optical microscopy; cf. Figure 4 C and Methods for further details. Note that, in these experiments, the DBCO-NP resides on the inside of the nanopipette, while the preprepared DNA/MP complex is present on the outside.

Accordingly, at *V*_bias_ = −1 V, the DNA/MP complex is driven into the pipet tip and particle tracking analysis reveals that the MP resides very close to the tip end. Upon bias reversal to +1 V, the MP moves away from its original position by on average 1.2 ± 0.25 μm but does not altogether leave the tip region, due to successful tethering of the DNA to the DBCO-NP on the inside of the pipet. The maximum tether length is shorter than the contour length of 5 kbp DNA (for B-type DNA, ≈ 1.7 μm),^28^ suggesting that the DNA is not fully extended in the weakening electric field outside of the nanopipette.^35–38^

In conclusion, we have demonstrated a robust nanopore-based strategy for the stepwise assembly and permanent trapping of DNA-AuNP dumbbell structures using quartz nanopipettes with sub-30 nm apertures. By taking advantage of directed, electric-field-driven assembly, we achieved controlled, multistep formation of NP/DNA/NP constructs directly in the tip of nanopipettes, an approach that is readily extended to other types of resistive-pulse sensors. Real-time electric recordings revealed distinct NP/DNA insertion events with sustained reduction in the pore current and increased noise. Voltage-switching experiments confirmed the stable formation of these structures, which was further supported by complementary, microparticle-based optical measurements. The latter not only validated the mechanical integrity of the constructs but also provided direct proof that the trapped structures remained mobile and responsive to external electric fields. With the demonstration of the fundamental design strategy, DNA building blocks may be further modified using established enzymatic or self-assembly techniques, to include capture probes for specific biomolecular targets. Hence, the ability to repeatedly transport such functionalized NP/DNA/NP structures through the sensing region of the nanopore may then enable high-fidelity electric readout of target binding at the level of individual carrier molecules, overcoming important limitations of conventional resistive-pulse sensing. Our findings therefore establish a path to a novel, nanoelectromechanical sensing platform that may enable new modes of dynamic sensing and molecular interrogation, including in confined geometries and flow-based environments.

## Supplementary Material



## Notes

Further details on the synthesis and preparation of the DNA and the nanoparticle–DNA complexes, TEM imaging studies, nanopipette fabrication and characterization, additional translocation data sets for DNA and NP/DNA complexes as well as example current–time traces and events for relevant event classes, characterization results for the amplifier used as well as circuit simulations for translocation and insertion events, and additional translocation frequency data in the presence and absence of a NP/DNA/NP construct (PDF)

## †

Department of Medicinal Chemistry and Pharmacognosy, Faculty of Pharmacy, Jordan University of Science and Technology, P.O. Box 3030, Irbid 22110, Jordan

## Abbreviations

AC: alternating current
DBCO: dibenzocyclooctyne
DC: direct current
NEMD: nanoelectromechanical device
NP: nanoparticle
TEM: transmission electron microscopy

## References

[1] Ying Y.-L., Hu Z.-L., Zhang S., Qing Y., Fragasso A., Maglia G., Meller A., Bayley H., Dekker C., Long Y.-T. (2022). Nanopore-based technologies
beyond DNA sequencing. Nat. Nanotechnol..

[2] Movileanu L. (2009). Interrogating
single proteins through nanopores: challenges and opportunities. Trends Biotechnol..

[3] Howorka S., Cheley S., Bayley H. (2001). Sequence-specific
detection of individual
DNA strands using engineered nanopores. Nat.
Biotechnol..

[4] Meller A., Nivon L., Branton D. (2001). Voltage-Driven DNA Translocations
through a Nanopore. Phys. Rev. Lett..

[5] Goyal G., Freedman K. J., Kim M. J. (2013). Gold nanoparticle translocation dynamics
and electrical detection of single particle diffusion using solid-state
nanopores. Anal. Chem..

[6] Pitchford W. H., Kim H.-J., Ivanov A. P., Kim H.-M., Yu J.-S., Leatherbarrow R. J., Albrecht T., Kim K.-B., Edel J. B. (2015). Synchronized
Optical and Electronic Detection of Biomolecules Using a Low Noise
Nanopore Platform. ACS Nano.

[7] Zhao Y., Hubarevich A., De Fazio A. F., Iarossi M., Huang J.-A., De Angelis F. (2023). Plasmonic
Bowl-Shaped Nanopore for Raman Detection
of Single DNA Molecules in Flow-Through. Nano
Lett..

[8] Tang L., Nadappuram B. P., Cadinu P., Zhao Z., Xue L., Yi L., Ren R., Wang J., Ivanov A. P., Edel J. B. (2021). Combined
quantum tunnelling and dielectrophoretic trapping for molecular analysis
at ultra-low analyte concentrations. Nat. Commun..

[9] Albrecht T. (2012). Electrochemical
tunnelling sensors and their potential applications. Nat. Commun..

[10] Cecchini M. P., Wiener A., Turek V. A., Chon H., Lee S., Ivanov A. P., McComb D. W., Choo J., Albrecht T., Maier S. A. (2013). Rapid Ultrasensitive Single Particle Surface-Enhanced
Raman Spectroscopy Using Metallic Nanopores. Nano Lett..

[11] Wanunu M. (2012). Nanopores:
A journey towards DNA sequencing. Phys. Life
Rev..

[12] Rahman M., Sampad M. J. N., Hawkins A., Schmidt H. (2021). Recent advances
in
integrated solid-state nanopore sensors. Lab
Chip.

[13] da
Silva E. T. S. G., Souto D. E. P., Barragan J. T. C., de
Giarola F. J., de Moraes A. C. M., Kubota L. T. (2017). Electrochemical
Biosensors in Point-of-Care Devices: Recent Advances and Future Trends. ChemElectroChem.

[14] Fraccari R. L., Carminati M., Piantanida G., Leontidou T., Ferrari G., Albrecht T. (2016). High-bandwidth
detection of short
DNA in nanopipettes. Farad. Disc..

[15] Fraccari R. L., Ciccarella P., Bahrami A., Carminati M., Ferrari G., Albrecht T. (2016). High-speed
detection of DNA translocation
in nanopipettes. Nanoscale.

[16] Al-Waqfi R. A., Khan C. J., Irving O. J., Matthews L., Albrecht T. (2025). Crowding Effects
during DNA Translocation in Nanopipettes. ACS
Nano.

[17] Albrecht T. (2019). Single-Molecule
Analysis with Solid-State Nanopores. Annu. Rev.
Anal. Chem..

[18] Roelen Z., Tabard-Cossa V. (2023). Synthesis
of length-tunable DNA carriers for nanopore
sensing. PLoS One.

[19] Bell N. A. W., Keyser U. F. (2015). Specific Protein
Detection Using Designed DNA Carriers
and Nanopores. J. Am. Chem. Soc..

[20] Zhang X., Luo D., Zheng Y.-W., Li X.-Q., Song J., Zhao W.-W., Chen H.-Y., Xu J.-J. (2022). Translocation of Specific DNA Nanocarrier
through an Ultrasmall Nanopipette: Toward Single-Protein-Molecule
Detection with Superior Signal-to-Noise Ratio. ACS Nano.

[21] Bell N. A. W., Keyser U. F. (2016). Digitally encoded DNA nanostructures for multiplexed,
single-molecule protein sensing with nanopores. Nat. Nanotechnol..

[22] Albrecht T. (2017). Progress in
single-biomolecule analysis with solid-state nanopores. Curr. Op. Electrochem..

[23] Loh A. Y. Y., Burgess C. H., Tanase D. A., Ferrari G., McLachlan M. A., Cass A. E. G., Albrecht T. (2018). Electric Single-Molecule
Hybridization
Detector for Short DNA Fragments. Anal. Chem..

[24] Roelen Z., Briggs K., Tabard-Cossa V. (2023). Analysis of Nanopore Data: Classification
Strategies for an Unbiased Curation of Single-Molecule Events from
DNA Nanostructures. ACS Sens..

[25] Gershow M., Golovchenko J. A. (2007). Recapturing
and trapping single molecules with a solid-state
nanopore. Nat. Nanotechnol..

[26] Luo L., German S. R., Lan W.-J., Holden D. A., Mega T. L., White H. S. (2014). Resistive-Pulse
Analysis of Nanoparticles. Annu. Rev. Anal.
Chem..

[27] Gibb T. R., Ivanov A. P., Edel J. B., Albrecht T. (2014). Single Molecule Ionic
Current Sensing in Segmented Flow Microfluidics. Anal. Chem..

[28] King T. L., Gatimu E. N., Bohn P. W. (2009). Single nanopore transport of synthetic
and biological polyelectrolytes in three-dimensional hybrid microfluidic/nanofluidic
devices. Biomicrofluidics.

[29] Bell N. A. W., Engst C. R., Ablay M., Divitini G., Ducati C., Liedl T., Keyser U. F. (2012). DNA Origami
Nanopores. Nano Lett..

[30] Wang H.-Q., Deng Z.-X. (2015). Gel electrophoresis
as a nanoseparation tool serving
DNA nanotechnology. Chin. Chem. Lett..

[31] Pellegrino T., Sperling R. A., Alivisatos A. P., Parak W. J. (2007). Gel Electrophoresis
of Gold-DNA Nanoconjugates. J. Biomed. Biotechnol..

[32] Knowles S. F., Weckman N. E., Lim V. J. Y., Bonthuis D. J., Keyser U. F., Thorneywork A. L. (2021). Current
Fluctuations in Nanopores Reveal the Polymer-Wall
Adsorption Potential. Phys. Rev. Lett..

[33] Wen C., Zeng S., Arstila K., Sajavaara T., Zhu Y., Zhang Z., Zhang S.-L. (2017). Generalized
Noise Study of Solid-State
Nanopores at Low Frequencies. ACS Sens..

[34] Storm A. J., Storm C., Chen J., Zandbergen H., Joanny J.-F., Dekker C. (2005). Fast DNA Translocation through a
Solid-State Nanopore. Nano Lett..

[35] Smith S. B., Cui Y., Bustamante C. (1996). Overstretching
B-DNA: The Elastic Response of Individual
Double-Stranded and Single-Stranded DNA Molecules. Science.

[36] Ferree S., Blanch H. W. (2003). Electrokinetic Stretching
of Tethered DNA. Biophys. J..

[37] Ando G., Hyun C., Li J., Mitsui T. (2012). Directly Observing
the Motion of DNA Molecules near Solid-State Nanopores. ACS Nano.

[38] Friedrich S. M., Liu K. J., Wang T.-H. (2016). Single
Molecule Hydrodynamic Separation
Allows Sensitive and Quantitative Analysis of DNA Conformation and
Binding Interactions in Free Solution. J. Am.
Chem. Soc..

