# Supplementary material for: Constructing a Nanopipette-Based DNA Electromechanical Device
Source: Nano Lett. 2025 Dec 15;25(51):17852–9. doi: 10.1021/acs.nanolett.5c05156 (PMC12751022; doi:10.1021/acs.nanolett.5c05156)
Supplement: Supplementary file 1 [file nl5c05156_si_001.pdf]

Constructing a Nanopipette-based DNA Electro-Mechanical Device

*Cengiz J. Khan,[a] Oliver J. Irving,[a] Rand A. Al-Waqfi,[a] Giorgio Ferrari,[b] Tim Albrecht[a]**

[a]University of Birmingham, School of Chemistry, Edgbaston Campus, Birmingham B15 2TT, United Kingdom

[b]Department of Physics, Politecnico di Milano, P.za L. da Vinci 32, Milano, 20133, Italy

Supporting Information

S1. Synthesis of end-functionalised 5 kbp DNA

Primer sequences are provided in Table S1a. A PCR reaction was assembled using: 2.5 μL of 10 μM forward primer, 2.5 μL of reverse primer (IDT), 1 μL of 1 ng/μL lambda DNA (Sigma Aldrich), 19 μL of nuclease-free water (VWR), and 25 μL of 2X Q5® Hot Start Hi-Fidelity Master Mix (New-England Biolabs). The mixture was prepared on ice and run on a PrimeG thermocycler (Cole Parmer) under cycling conditions listed in Table S1b. The PCR product was purified using a DNA cleanup kit (Monarch® Spin PCR & DNA Cleanup Kit, New-England Biolabs).

DNA samples were analysed by agarose gel electrophoresis. A 1% gel was prepared by dissolving 1g agarose in 100 mL 1X TAE buffer (VWR), heating to 70°C, and pouring into a casting tray. Samples (5 μL DNA + 1 μL TriTrack loading dye (Thermo Fisher)) and 5 μL GeneRuler 1 kb ladder (Thermo Fisher) were loaded and run at 80 V for 45 minutes. Gels were stained with 1X SYBR Gold (Invitrogen) for 45 minutes and imaged using a UV Vis illuminator (GelDoc Go system, Bio-Rad). DNA concentrations were determined using a NanoDrop spectrophotometer (Shimadzu) by measuring absorbance at 260nm.

The sequences and PCR cycling parameters are shown in Table S1a and Table S1b respectively.

Table S1a - Primer sequences

Name	Sequence (5' – 3')	5' Modification
Forward Primer	ATTTACAGCGGCAGCCATAAGGT	Biotin
Reverse Primer	TCATCAGGGCGAGATGCTCAATG	Azide

Table S1b – PCR cycling parameters

STEP	TEMP/°C	TIME/s
Initial Denaturation	98	30
30 Cycles	98<br>70<br>72	10<br>20<br>150
Final Extension	72	120
Hold	5	-

S2. DNA-AuNP conjugation

Purified 5 kbp DNA was diluted to a final concentration of 11.6 nM in nuclease-free water. A 60 μL aliquot of this DNA was mixed with 60 μL of 11.6 nM DBCO-functionalised AuNPs (Nanopartz Inc.). The reaction was incubated at room temperature for 18 hours on a shaker to allow copper-free click conjugation via DBCO-azide linkage. The resulting reaction mixture was used directly for translocation and trapping experiments. From the integration of bands in gel shift assays, we estimate the average yield to be 40-60%.

For the agarose gel and TEM imaging experiments (main text, fig. 2), the full construct was prepared in solution. For this purpose, 60 μL of 11.6 nM streptavidin-functionalised AuNPs (Nanopartz Inc.) were added to 60 μL of the DBCO-AuNPs/5 kbp DNA mixture. The reaction was incubated at room temperature for 30 minutes on a shaker to allow for biotin-avidin binding. This enabled the formation of both half constructs (DNA with a single AuNP) and full dumbbell structures (DNA tethered to two AuNPs), allowing further structural characterisation. More examples of TEM imaging of full dumbbell structures are shown in fig S1.

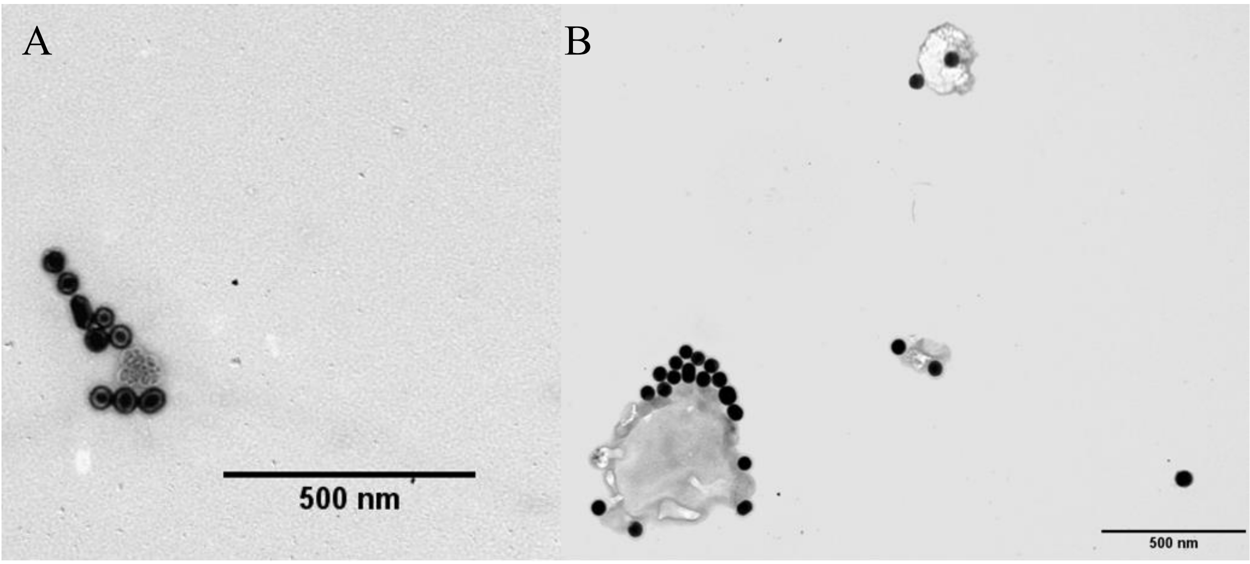

Figure S1 Further TEM imaging of NP/DNA/NP dumbbell structure mixtures acquired using JEOL JEM-1400 transmission electron microscope at 80 kV (Panels A and B). Dumbbell-like assemblies are visible among regions of particle clustering and DNA agglomeration, likely arising from the drop-casting process. While the overall morphology remains well preserved, localized beam-induced degradation of the lighter DNA regions is observed in panel B.

S3. Particle size distribution

A 1 µL aliquot of 0.1 nM AuNPs was drop-cast onto a copper TEM grid (Electron Microscopy Sciences, Pennsylvania, USA) and left to dry for 1 h under ambient conditions. The grid was subsequently washed with 10 µL of distilled water to remove residual impurities and dried for an additional 1 h. Imaging was performed using a JEOL 1400 transmission electron microscope, and representative micrographs are shown in fig. S1a. The micrograph was then analyzed using ImageJ to obtain particle size distribution as shown in fig. S2b.

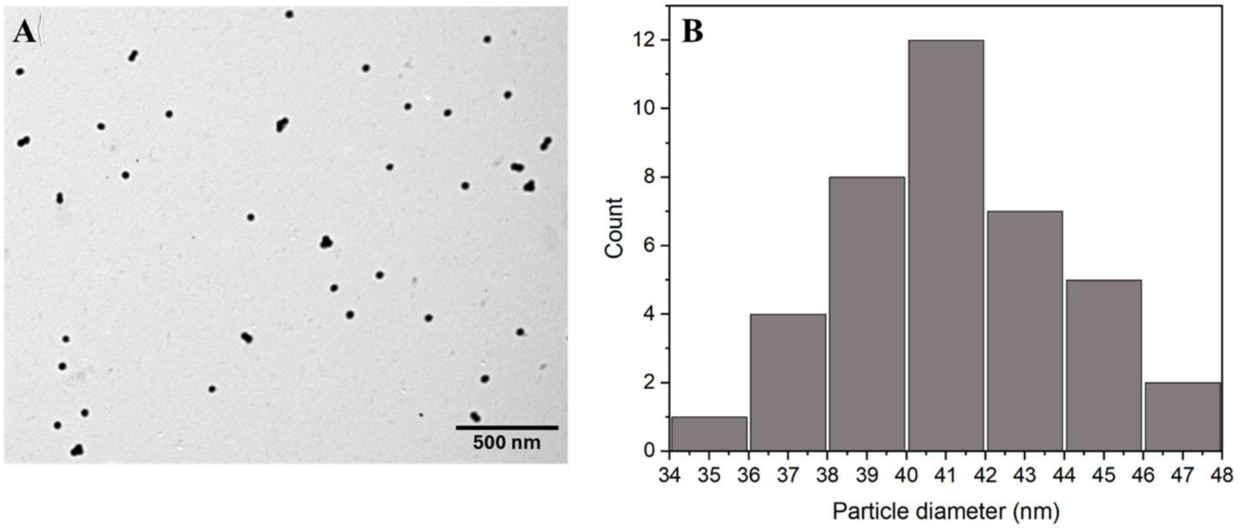

Figure S2 A sample of deposited AuNPs was imaged using a JEOL JEM-1400 transmission electron microscope at 60kV. Approximately 40 particles were manually measured (horizontally) using ImageJ software. The resulting histogram showed an approximately normal distribution centered at 41 nm ± 3.7 nm, consistent with vendor specifications of 40-42 nm.

S4. Nanopipette fabrication and electrical characterisation

Quartz capillaries (1.00 mm OD, 0.50 mm D_i, 7.5 cm length; World Precision Instruments) were plasma-cleaned for 5 minutes and pulled into nanopipettes using a P-2000 laser puller (Sutter Instruments) with Program 59 (parameters in Table S2a). The resulting pipettes were imaged under a Nikon Eclipse Ti2 optical microscope to measure taper lengths of the nanopipettes. The taper length is defined as the length from the tip of the capillary to where the internal channel had reached D_i. Ag/AgCl electrodes were made by cutting 10 cm of silver wire (0.25 mm diameter, 99.99% purity, Goodfellow) and immersed in 38% v/v nitric acid (Sigma Aldrich) for 10 s, then washed with Milli-Q water (18 MΩ, Merck Millipore) to remove surface impurities. The cleaned wires were soldered to gold contact pins and submerged in 4 M LiCl 1xTE solution. Annodization

was performed in an electrochemical cell using a gold wire (99.99% purity, Goodfellow) as a counter electrode and applying a current of 1 mA for 5 minutes, until the electrode surface turned black.

I-V measurements were performed using a CompactStat potentiostat (Ivium Technologies) to calculate the pore conductance, G. Taking the ionic conductivity g(c) for 4 M LiCl to be 173 mS cm^{-1}, the pore diameter (d_{pore}) was estimated from G, the inner diameter of the capillary, D_i = 1 mm, and the taper length (l) using Equation 1.[1]

$$d_{pore} = \frac{4Gl + \frac{\pi}{2}GD_i}{D_i \pi g(c) - \frac{\pi}{2}G} \qquad (1)$$

A summary of pipette geometries and conductance-derived diameters is shown in Table S2b. From this set, pipette 1 was selected for the translocation of unbound dsDNA (control), while pipettes 2 and 6 were used for all device experiments.

Table. S2a P-2000 pull parameters (Programme 59)

Programme 59					
Line	Pull	Heat	Filament	Velocity	Delay
1	75	700	5	35	150
2	200	700	0	15	128

Table. S2b: Sample of 10 nanopipettes prepared under the same experimental conditions described in the main text, inc. taper length, conductance and estimated pore diameter d_{pore}.

Pipette	Taper length (µm)	Conductance (nS)	Pore diameter (nm)
1*	3060	38	19
2**	3125	47	24
3	3400	26	14
4**	3263	48	25
5	2834	74	33
6	3119	50	24
7	3324	27	14
8	2987	51	24
9	3245	40	18
10	3256	34	14
Mean	3161	44	21
SD	170	14	6

*Pipette used for unbound dsDNA control experiments

**Pipette used for DNA-AuNP device measurements, see fig. 3 in the main manuscript (pipette 2) and fig. S3b below (pipette 4).

S5 DNA translocation and trapping experiments

DNA was injected separately into the bulk solution in a 3 mL liquid cell containing 2 mL 4 M LiCl 1XTE to a final concentration of DNA ~300 pM. The liquid cell was housed in a double Faraday cage to reduce electrical interference. A negative bias value means that the electrode outside the nanopipette is biased negatively, thereby resulting in an electrophoretic driving force for (negatively charged) DNA to translocate into the pipette.

Experiments were conducted in a semiautomated fashion using in-house MATLAB code with a sequence of applied biases, where for each bias, 102 data files of 10 s each were collected before the next bias value was applied.

Data recording was performed at a sampling rate of 1 MHz using a custom-built low-noise, high-bandwidth amplifier connected to the digital oscilloscope for analogue-to digital conversion (Picoscope 4262 Pico Technology), as reported previously.[1-4] Briefly, in this design, the input current is split into two output channels, namely the ‘’DC’’ and ‘’AC’’ channels. The former contains slow modulations of the input current (cutoff frequency ~ 7 Hz), including the open pore current. The AC channel contains fast modulations of the input current, for example, (short-lived) standard translocation events, and usually is zero mean, facilitating baseline correction.

Using custom-built MATLAB code, events were detected with a $5\sigma_{AC}$ threshold, where σ_{AC} was the standard deviation of the noise in the AC channel. For each detected event, relevant segments of the current-time trace were extracted from and up to the adjacent zero crossings and relevant event characteristics were determined, such as the event duration based on 1σ threshold crossings (τ), which we found to better capture the characteristics of events with complex shapes. Additionally, the effective current values (ΔI_e) of the events were calculated by dividing the event charge deficit (q) by event duration (τ). Where, event charge (q) is the integral of the current signal over the duration of the detected event[1, 3-5].

Finally, to calculate the change in average I_{DC} (ΔI_{DC}) and in the AC channel noise ($\Delta\sigma_{AC}$) we recorded the I_{DC} and $\Delta\sigma_{AC}$ values at 100 ms time windows before and after the event ends in the AC channel.

S6 Translocation of DNA and NP-DNA at different V_{bias} and other controls

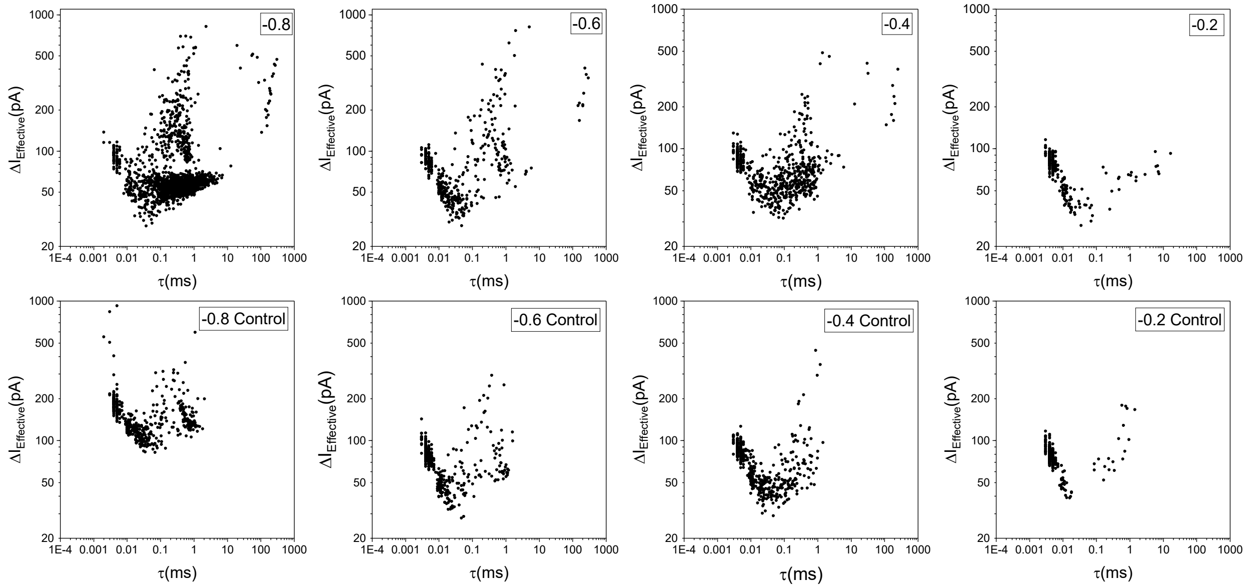

Figure S3a. Scatter plots of ΔI_e vs. τ for AuNP–DNA mixtures and DNA-only control samples recorded across applied biases (±0.2 to ±0.8 V). Both datasets were obtained using 300 pM PCR-amplified DNA, with control experiments performed in the absence of AuNPs in the electrochemical cell. Each scatter plot corresponds to a continuous 1000 s recording prior to switching to the next bias condition. The datasets at V_{bias} = -0.8 V are already shown in fig. 3 A/B.

The following results are from experiments similar to those reported in fig. 3 of the main manuscript, showing i.a. insertion of NP/DNA constructs into a nanopipette (V_{bias} =-0.8 V, 4 M LiCl electrolyte, with nanopipette 4 in Table S2b).

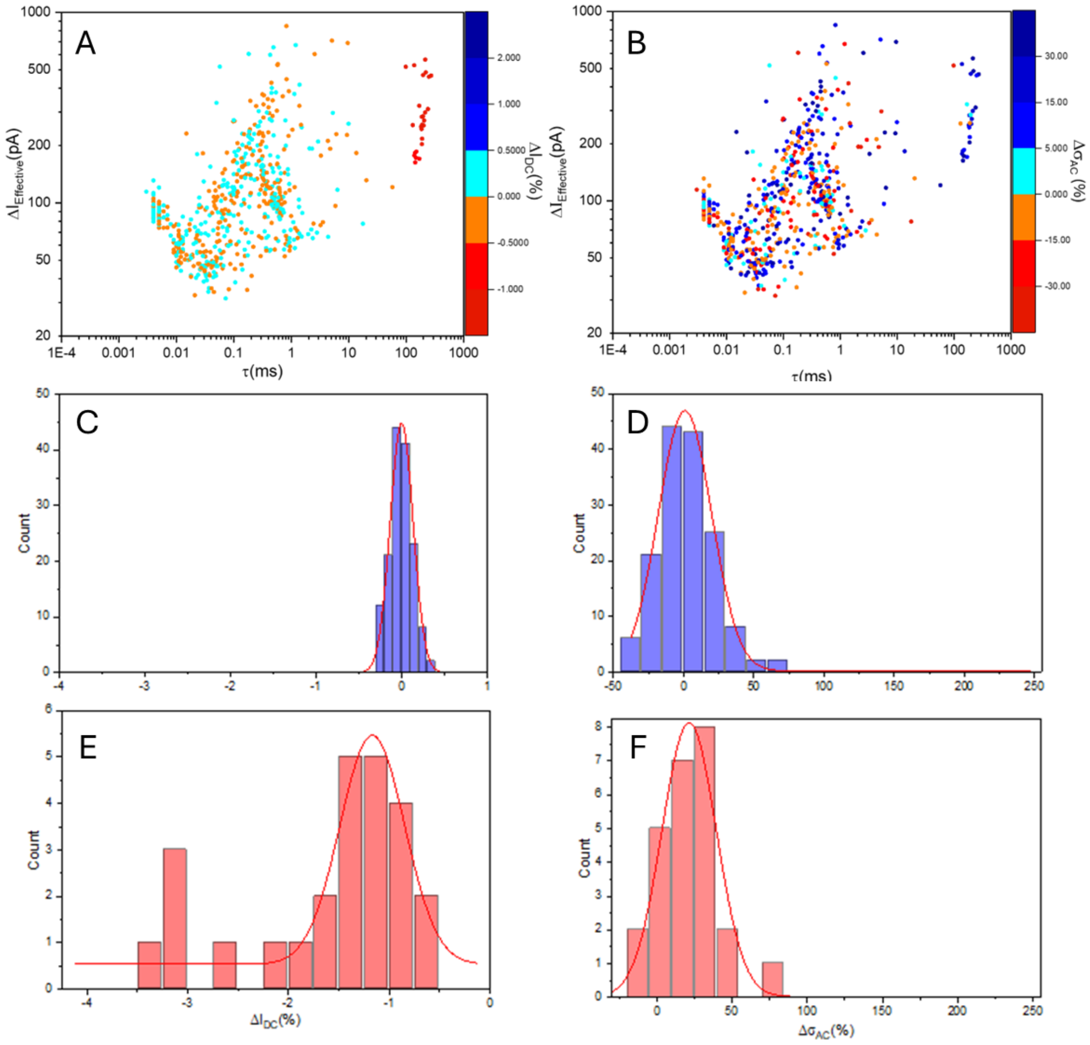

Figure S3b, A/B**:** Scatter plots for an insertion experiment, as described in the main text, but with nanopipette 4. Colour-coding according to ΔI_{DC} and $\Delta\sigma_{AC}$ values as in fig. 3. C-F: Histogram data for conventional DNA translocaton experiments (C/D) and insertion events (E/F). Insertion events are again characterized by a decrease in ΔI_{DC} of ~ 1.2% and an increase in $\Delta\sigma_{AC}$ of ~ 20%, similar to the results reported in the main text. Both were found to be statistically significantly different at a significance level $\alpha = 0.05$ (Mann-Whitney, $p = 1\times10^{-30}$ and $p = 3\times10^{-6}$, respectively).

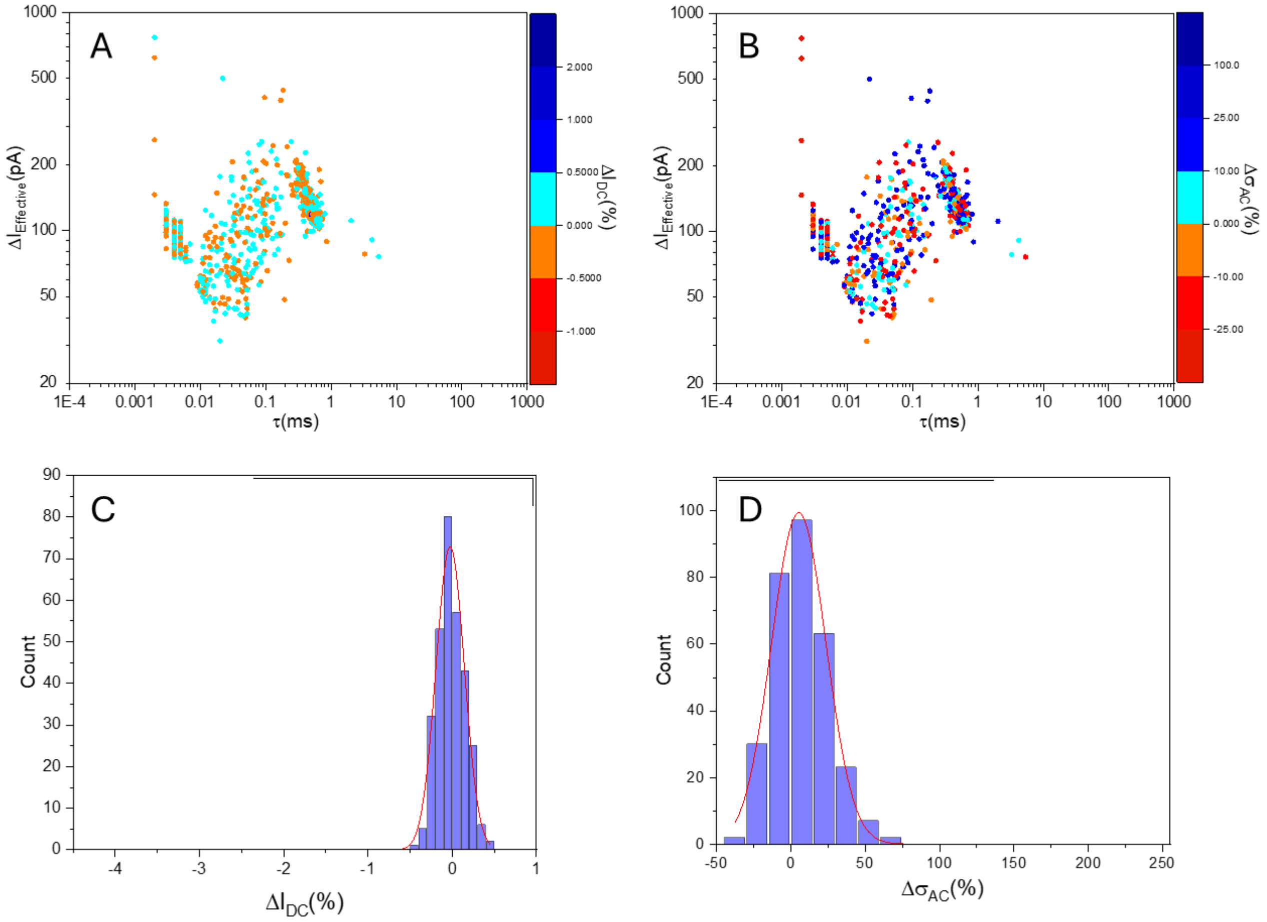

Figure S3c: A/B Data from fig. 3A, now with colour-coding of ΔI_{DC} and $\Delta\sigma_{AC}$, for comparison. C/D: histogram data of ΔI_{DC} and $\Delta\sigma_{AC}$ for the DNA translocation cluster. While there is some random fluctuation of these quantities, both are close to zero as expected for transient translocation events.

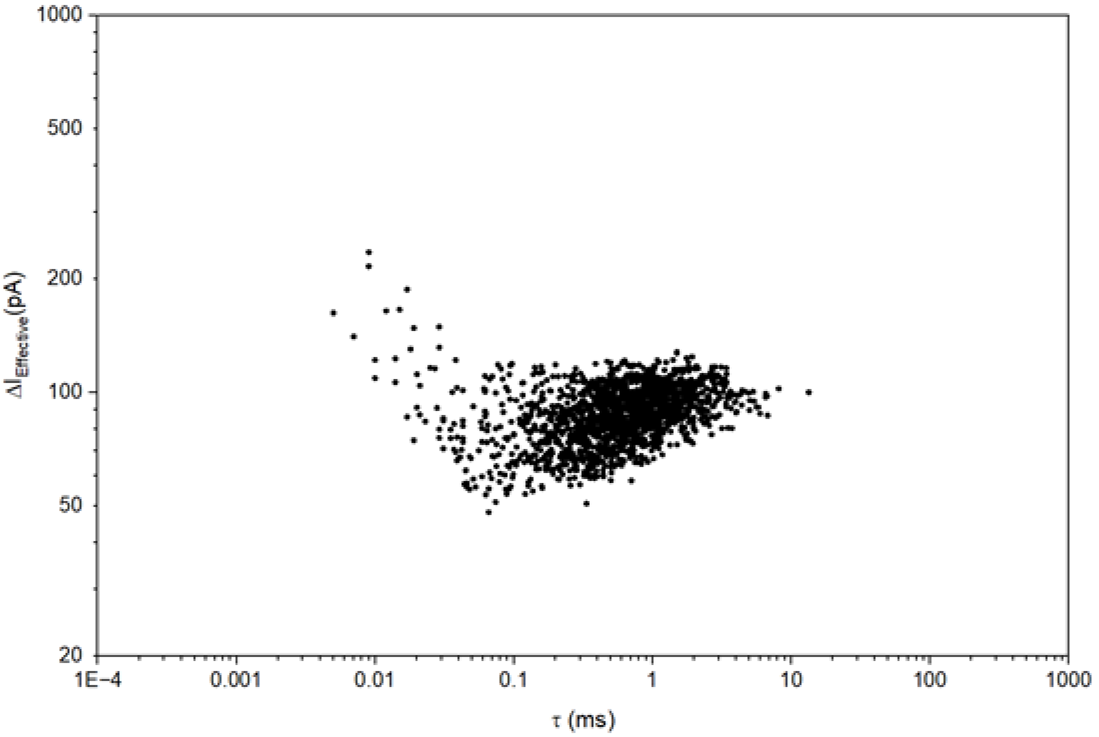

Fig. S3d: Translocation of 40 nm, DBCO-modified Au nanoparticles (V_{bias} = -0.8 V, 4 M LiCl, c_{NP} + 250 pM (outside only); d_{pore} = 28 nm). The above cluster is reminiscent of a similar feature in the NP/DNA insertion experiments, cf. fig. 3B and S3a. However, given their small positive charge, the applied bias is expected to exert a force that moves the particles away from the nanopipette entrance. Combined with the relatively long duration and large variance of the event time, it is unlikely that these events correspond to conventional translocation events and rather reflect a more complex interaction between the particles and the nanopipette.

S7 Example current-time traces from translocation experiments (AC channel)

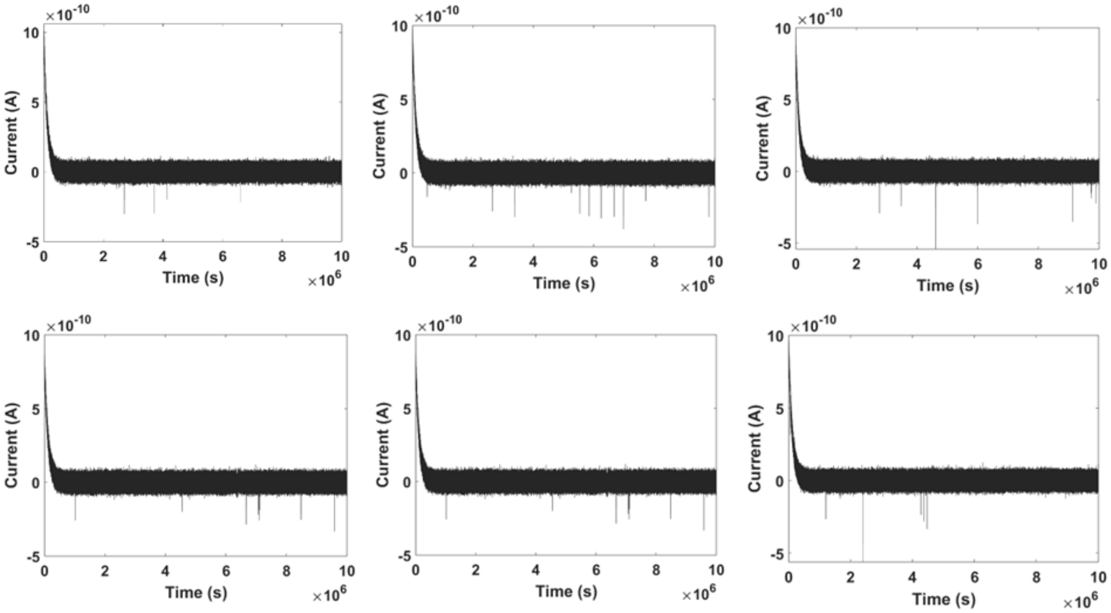

Figure S4a. Translocation of PCR products, six randomly chosen current-time traces (10 s each), cf. fig, 3A (V_{bias} = -0.8 V).

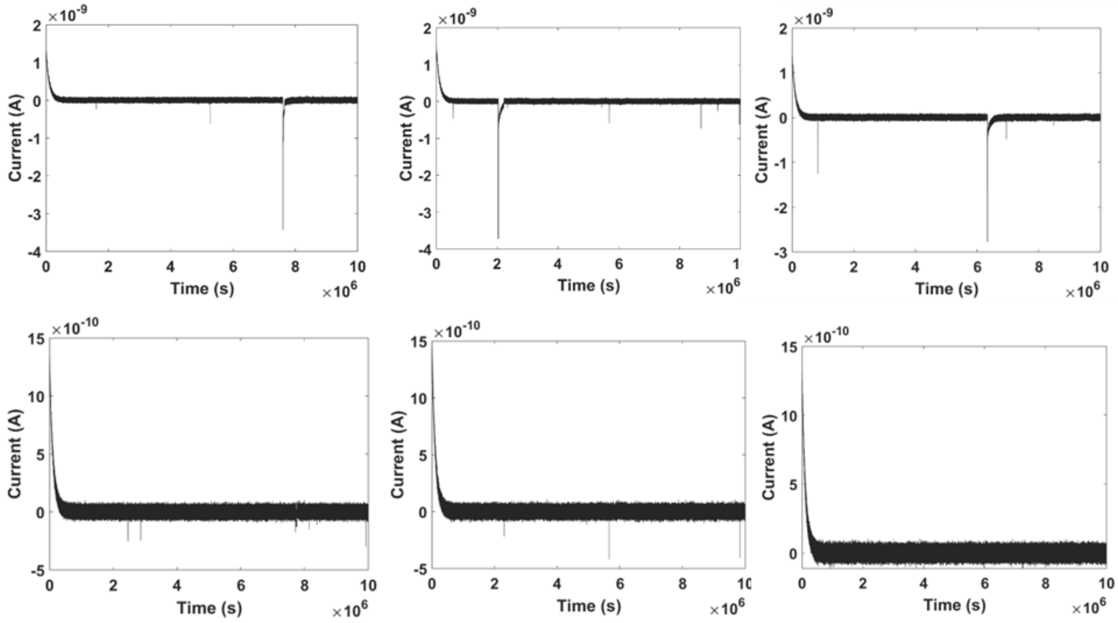

Figure S4b. Translocation experiment involving NP/DNA insertion, six randomly chosen current-time traces (10 s each). Due to stochastic fluctuations, the final trace does not contain any event.

S8 Examples of individual events (AC channel), from the dataset shown in fig. 3B

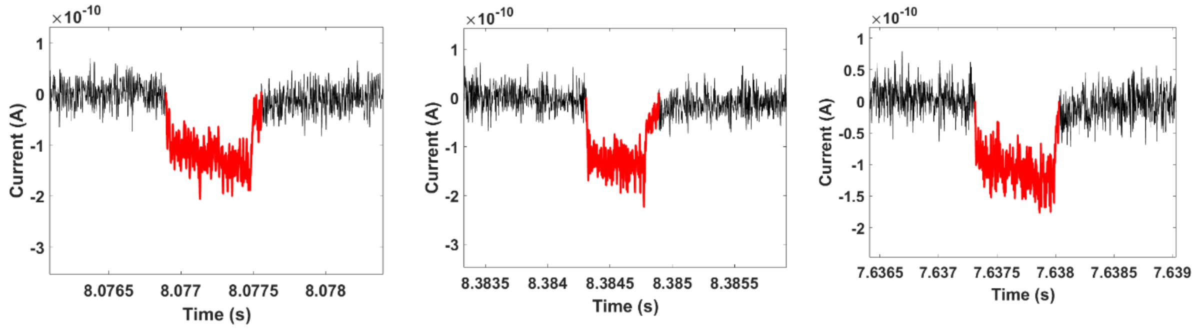

Figure S5a. Linear DNA events

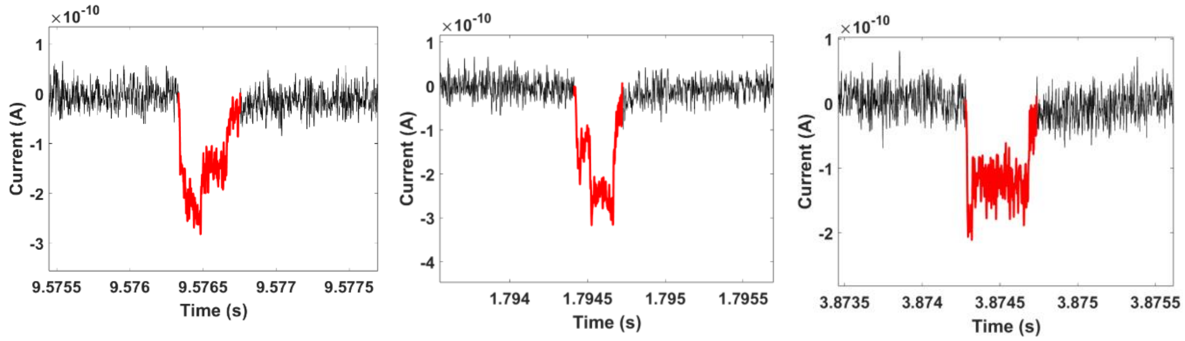

Figure S5b. Non-linear (folded) DNA events

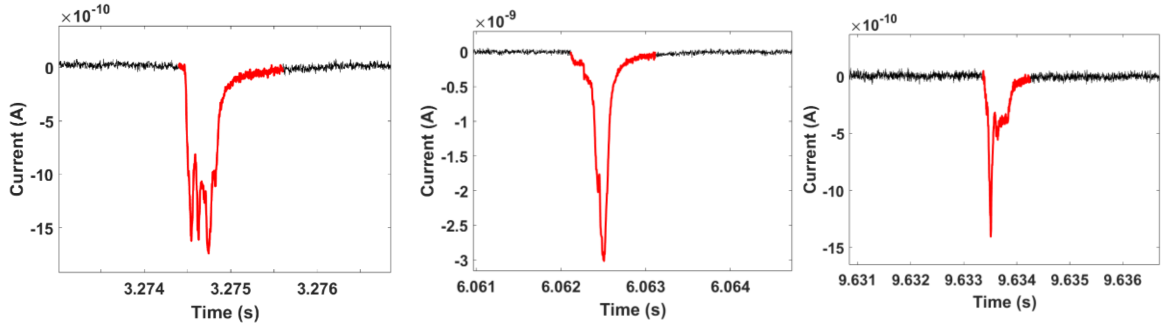

Figure S5c. Intermittent insertion NP/DNA events

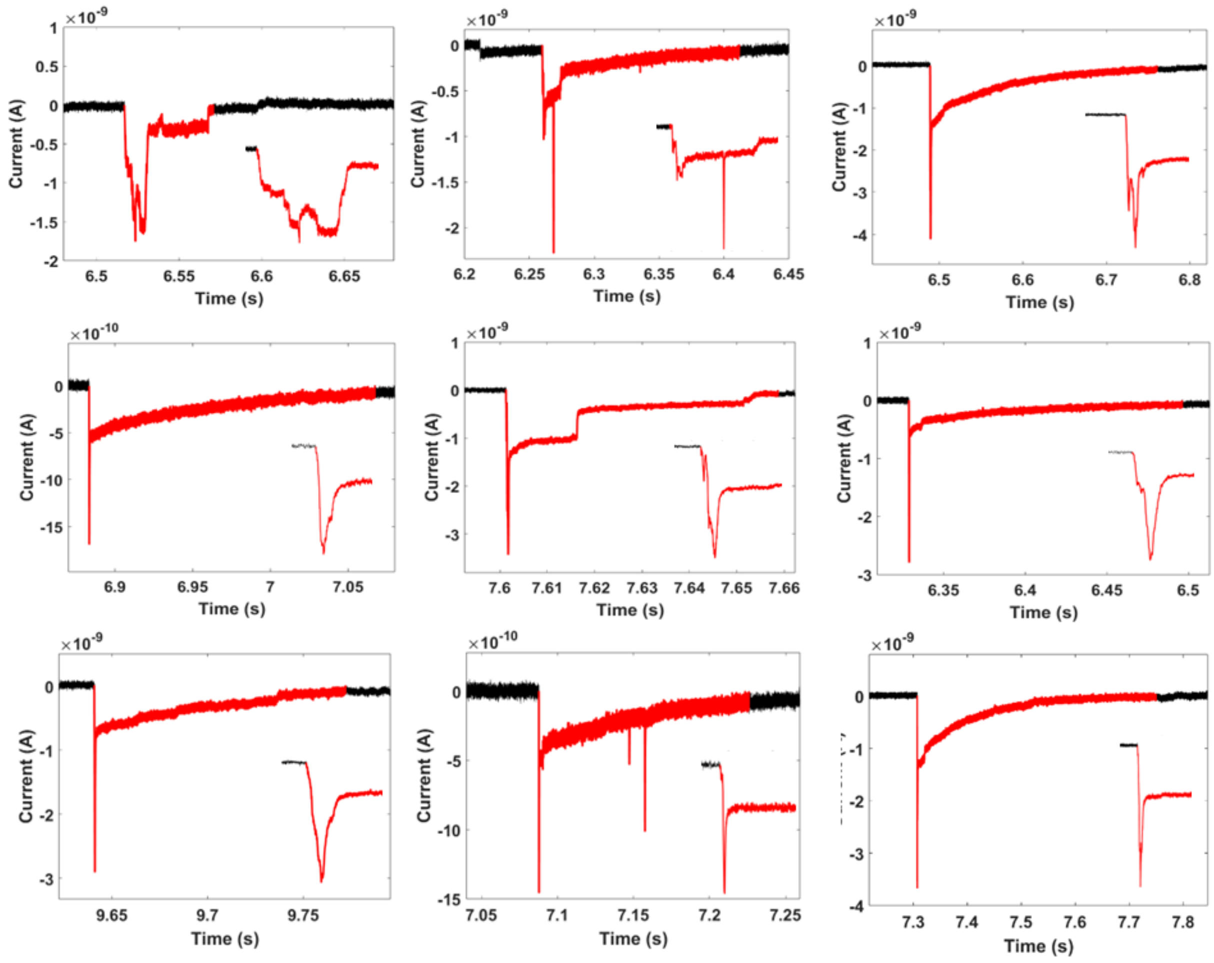

Figure S5d. Representative insertion events for AuNP–DNA at –0.8 V. Traces show current blockades exceeding 1 nA followed by a gradual recovery of the AC channel current to zero mean. Several events display sub-features consistent with initial particle–pore collisions, highlighted in the insets.

S9 – CMOS current amplifier

The current I_{IN} from the nanopipette is measured using the custom amplifier shown in fig. S6.

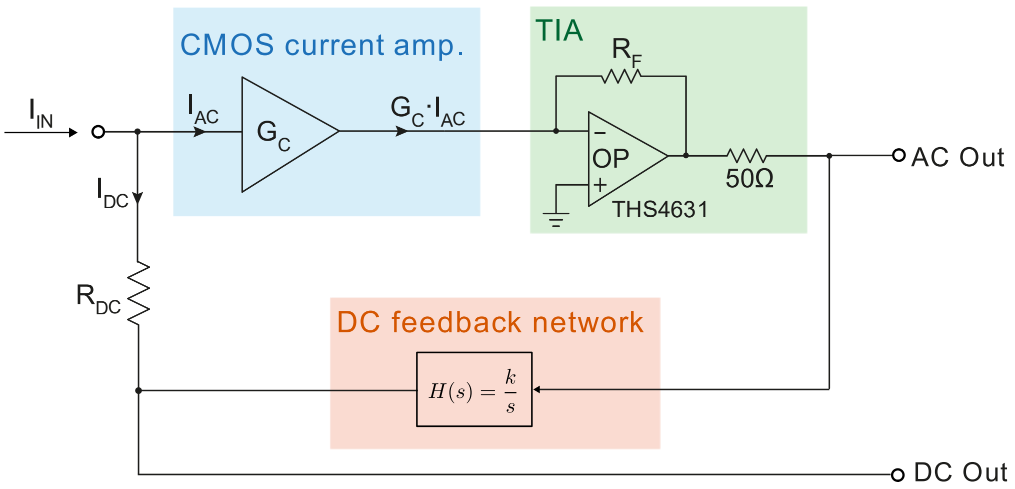

Figure. S6: Simplified scheme of the custom amplifier with two output channels.

It is based on a low-noise wide-bandwidth CMOS current amplifier with a gain $G_C = 990$ and bandwidth of 1MHz.[6] The amplified current is converted into a voltage by a standard transimpedance amplifier with a feedback resistor of $R_F = 51$ kΩ. Since the noise of the CMOS current amplifier is proportional to the DC current at its input, an additional feedback network H(s) operating at low frequencies forces the stationary input current into the off-chip resistor $R_{DC} = 100$ MΩ. H(s) is made by an integrator stage (k ≅ 20 s^{-1}) whose gain decreases with frequency, deactivating the feedback loop at high frequency and allowing the current amplifier to amplify the fast input current variations. As a result, the voltage across the resistor R_{DC} (DC output) is a low-pass filtered version of the input current I_{IN} given by equation 2.

$$V_{out,DC} = \frac{R_{DC}}{1+s\tau_{DC}} I_{IN} \qquad (2)$$

where τ_{DC}= $1/2\pi f_{DC}$= R_{DC} /($G_{DC}\cdot R_F\cdot k$) ≅ 22 ms is the time constant of the feedback loop. On the contrary, the TIA output (AC output) is a high-pass filtered version of the input current in equation 3:

$$V_{out,AC} = G_c\, R_F \frac{s\tau_{DC}}{(1+s\tau_{DC})(1+s\tau_H)}\; I_{IN} \qquad (3)$$

where τ_H is the time constant of the high-frequency pole (≈ 2 MHz) that limits the overall bandwidth of the custom amplifier and is given by the TIA.

The simulated step response of the amplifier is reported in Fig. S7 in the case of an abrupt variation from 1 nA to 800 pA. The outputs of the amplifier are reported divided by the nominal gains, i.e., R_{DC} for the DC channel and $G_C\cdot R_F$ for the AC channel. As expected, the DC channel maintains the information on the mean value of the input current and follows the input variation with a settling time of about 100 ms. The AC channel correctly measures the fast variation of the input current on a time scale shorter than the response time of the DC feedback loop, τ_{DC}. On a longer time scale, the input current is increasingly forced by the DC feedback network to flow in R_{DC}, reducing the AC output and correspondingly changing the DC output.

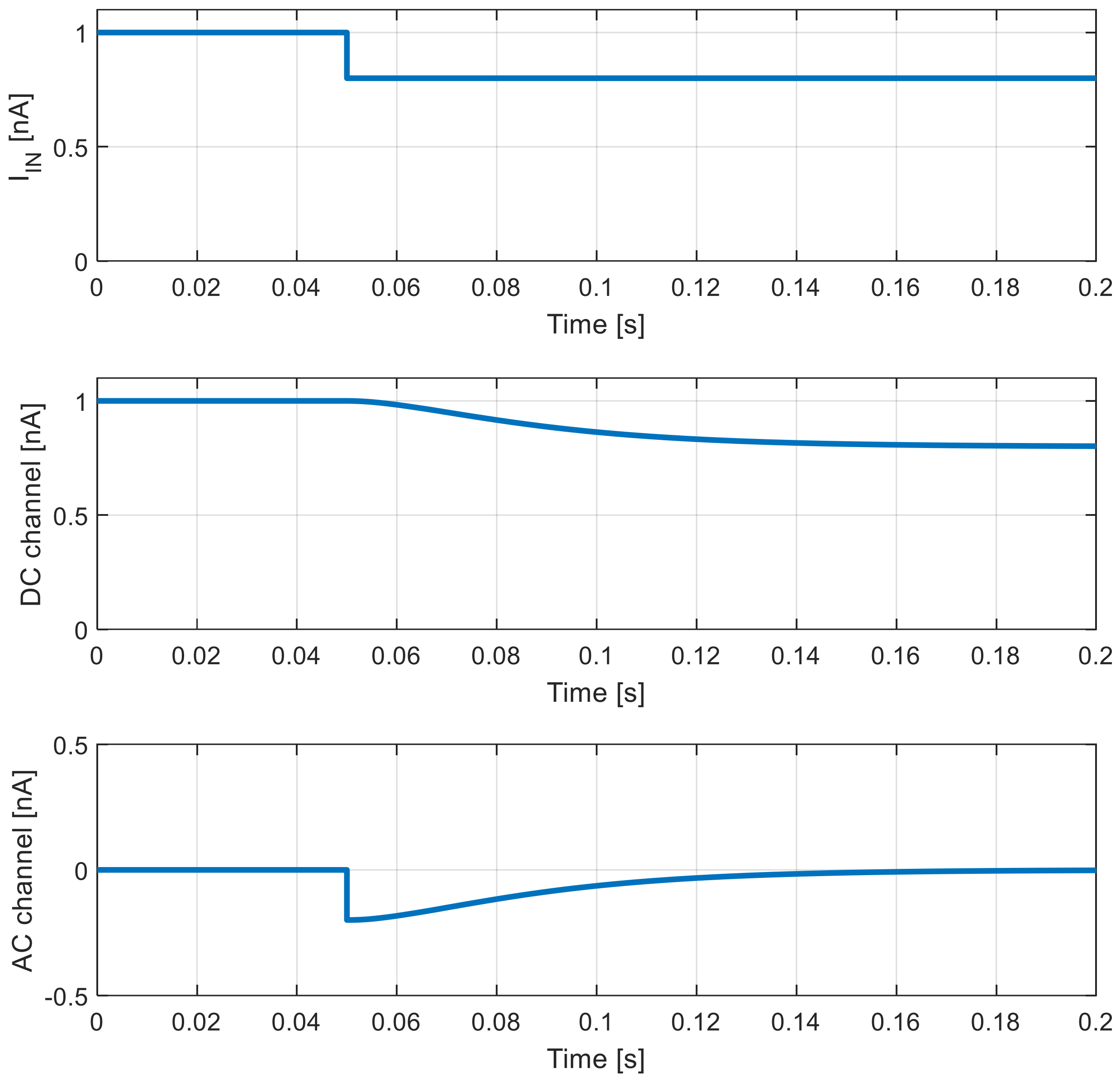

Figure. S7: Simulated response of the amplifier to a current step from 1nA to 800pA.

Fig. S8 reports the simulated response of the amplifier to a current pulse with a duration of 1 ms. Since the pulse duration is lower than the response time of the feedback loop, $\tau_{DC} \cong 22$ms, the voltage across R_{DC} (i.e. the DC output) has no time to change significantly. Consequently, the current in R_{DC} remains constant during the pulse, allowing the CMOS current amplifier and the TIA to fully process the current variation, as shown by the AC channel output.

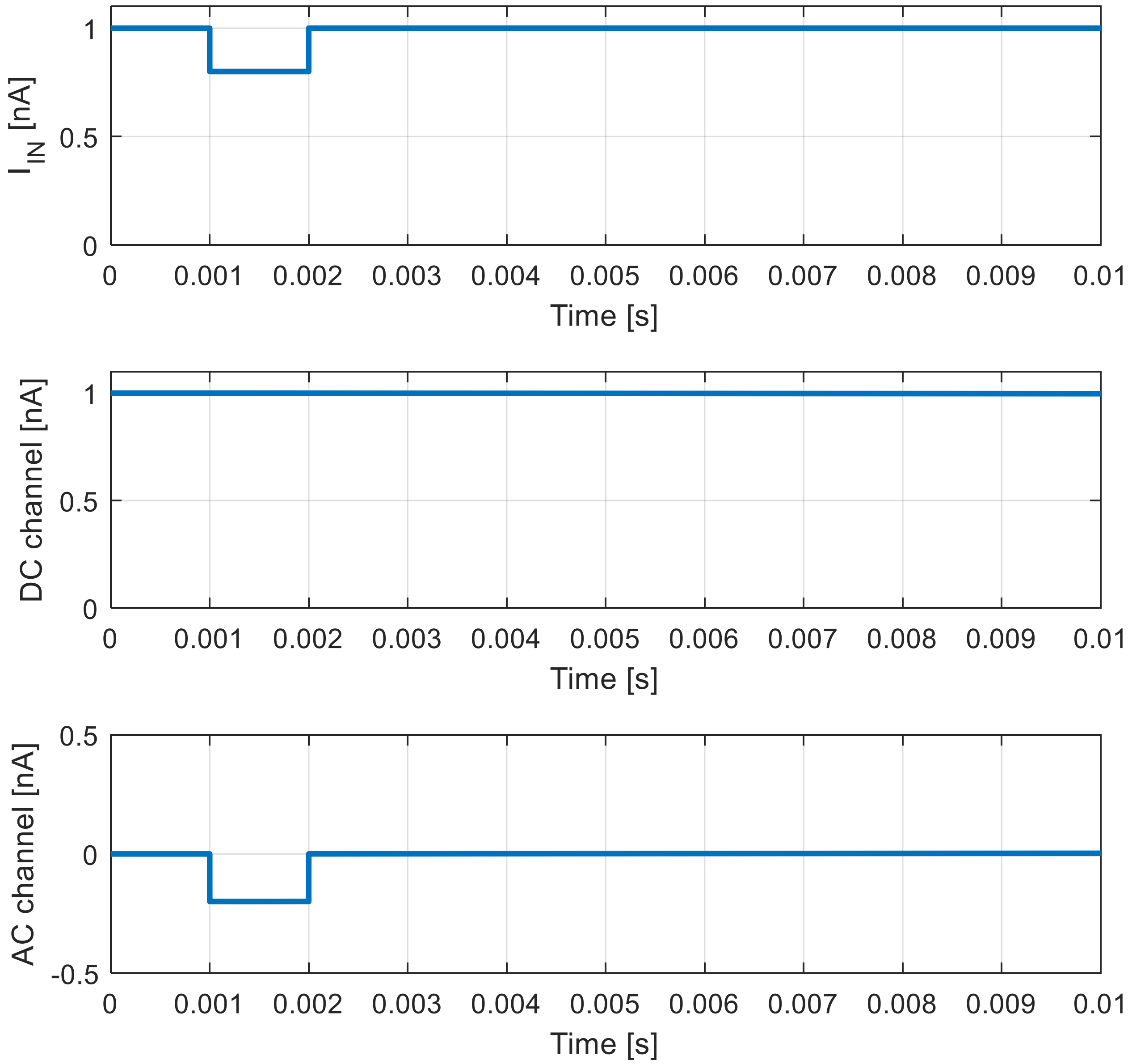

Figure. S8: Simulated response of the amplifier to a current pulse of 200pA and duration 1ms.

S10. Comparison of translocation frequencies, in the presence and absence of NP/DNA/NP

The translocation of free dsDNA continued in the presence of the NP/DNA/NP construct, albeit at a significantly reduced frequency. Fig. S9 below shows the probability distribution of translocation frequencies in the two cases. Even though the nanopore is smaller in the control experiment ($d_i \approx$ 19 nm vs. 24 nm), the mean translocation frequency is larger than for the nanopipette with the trapped NP/DNA/NP construct.

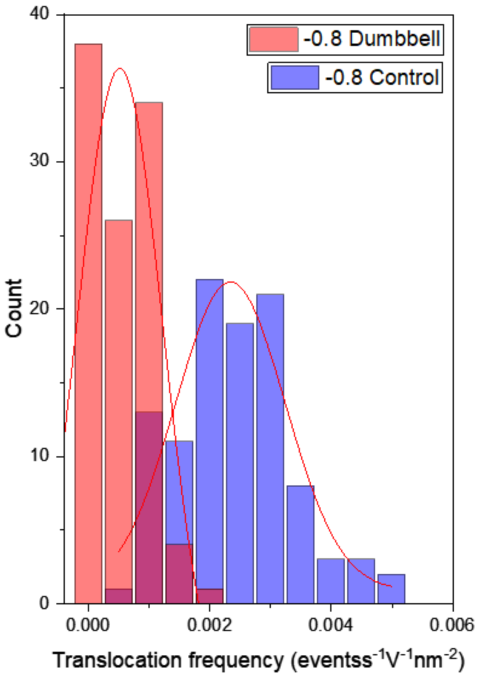

Figure. S9 shows normalised translocation frequencies of 5 kbp DNA for two conditions: with the trapped NP/DNA/NP structure in place (red bars, $d_i \approx 24$ nm), and control (blue bars, $d_i \approx 19$ nm).

References

(1) Al-Waqfi, R. A.; Khan, C. J.; Irving, O. J.; Matthews, L.; Albrecht, T. Crowding Effects during DNA Translocation in Nanopipettes. *ACS Nano* **2025**, 19, 17, 16803–16812.

(2) Ying, Y.-L.; Hu, Z.-L.; Zhang, S.; Qing, Y.; Fragasso, A.; Maglia, G.; Meller, A.; Bayley, H.; Dekker, C.; Long, Y.-T. Nanopore-based technologies beyond DNA sequencing. *Nat. Nanotechnol.* **2022**, *17* (11), 1136-1146.

(3) Fraccari, R. L.; Carminati, M.; Piantanida, G.; Leontidou, T.; Ferrari, G.; Albrecht, T. High-bandwidth detection of short DNA in nanopipettes. *Farad. Disc.* **2016**, *193*, 459-470.

(4) Fraccari, R. L.; Ciccarella, P.; Bahrami, A.; Carminati, M.; Ferrari, G.; Albrecht, T. High-speed detection of DNA translocation in nanopipettes. *Nanoscale* **2016**, *8* (14), 7604-7611.

(5) Loh, A. Y. Y.; Burgess, C. H.; Tanase, D. A.; Ferrari, G.; McLachlan, M. A.; Cass, A. E. G.; Albrecht, T. Electric Single-Molecule Hybridization Detector for Short DNA Fragments. *Anal. Chem.* **2018**, *90* (23), 14063-14071.

(6) Ferrari, G.; Farina, M.; Guagliardo, F.; Carminati, M.; Sampietro, M. Ultra-low-noise CMOS current preamplifier from DC to 1 MHz. *Electron. Lett.* **2009**, *45* (25), 1278-1280.
